# Time to initial cancer treatment in the United States and association with survival over time: An observational study

**DOI:** 10.1371/journal.pone.0213209

**Published:** 2019-03-01

**Authors:** Alok A. Khorana, Katherine Tullio, Paul Elson, Nathan A. Pennell, Stephen R. Grobmyer, Matthew F. Kalady, Daniel Raymond, Jame Abraham, Eric A. Klein, R. Matthew Walsh, Emily E. Monteleone, Wei Wei, Brian Hobbs, Brian J. Bolwell

**Affiliations:** 1 Taussig Cancer Institute, Cleveland Clinic, Cleveland, OH, United States of America; 2 Digestive Disease and Surgery Institute, Cleveland Clinic, Cleveland, OH, United States of America; 3 Heart and Vascular Institute, Cleveland Clinic, Cleveland, OH, United States of America; 4 Glickman Urology and Kidney Institute, Cleveland Clinic, Cleveland, OH, United States of America; University of South Alabama Mitchell Cancer Institute, UNITED STATES

## Abstract

**Background:**

Delays in time to treatment initiation (TTI) for new cancer diagnoses cause patient distress and may adversely affect outcomes. We investigated trends in TTI for common solid tumors treated with curative intent, determinants of increased TTI and association with overall survival.

**Methods and findings:**

We utilized prospective data from the National Cancer Database for newly diagnosed United States patients with early-stage breast, prostate, lung, colorectal, renal and pancreas cancers from 2004–13. TTI was defined as days from diagnosis to first treatment (surgery, systemic or radiation therapy). Negative binomial regression and Cox proportional hazard models were used for analysis. The study population of 3,672,561 patients included breast (N = 1,368,024), prostate (N = 944,246), colorectal (N = 662,094), non-small cell lung (N = 363,863), renal (N = 262,915) and pancreas (N = 71,419) cancers. Median TTI increased from 21 to 29 days (P<0.001). Aside from year of diagnosis, determinants of increased TTI included care at academic center, race, education, prior history of cancer, transfer of facility, comorbidities and age. Increased TTI was associated with worsened survival for stages I and II breast, lung, renal and pancreas cancers, and stage I colorectal cancers, with hazard ratios ranging from 1.005 (95% confidence intervals [CI] 1.002–1.008) to 1.030 (95% CI 1.025–1.035) per week of increased TTI.

**Conclusions:**

TTI has lengthened significantly and is associated with absolute increased risk of mortality ranging from 1.2–3.2% per week in curative settings such as early-stage breast, lung, renal and pancreas cancers. Studies of interventions to ease navigation and reduce barriers are warranted to diminish potential harm to patients.

## Introduction

Delays in time to treatment initiation (TTI) for new cancer diagnoses are commonly known to cause patient anxiety and distress [1, 2, 3, 4]. Physicians often reassure patients that current wait times to initiate therapy will not impact long-term outcomes, but the evidence is conflicting. Studies in breast, head and neck, gynecologic, and lung cancer suggest that increased time to treatment initiation (TTI) is associated with worsened survival [5, 6, 7, 8, 9, 10]. Other studies, however, suggest no association of increased TTI on survival [10, 11, 12, 13, 14].

United States (US) health care providers perceive that TTI is worsening with increasing complexity of health-systems and requirements for prior authorizations by insurers [15, 16, 17, 18, 19]. Median wait times for surgery increased in the decade prior to 2005 [20].

We therefore conducted a comprehensive and contemporaneous analysis of TTI across early-stage solid tumors treated with curative-intent approaches in the US to address these knowledge gaps. The objectives were to investigate trends in TTI, identify determinants of increased TTI and to evaluate the relationship between TTI and overall survival using hospital-based data from the National Cancer Database (NCDB) for newly diagnosed breast, prostate, lung, colorectal, renal and pancreas cancer patients.

## Methods

The study cohort was obtained in de-identified form from the NCDB, a hospital-based, prospectively collected nationwide oncology outcomes database [21]. The NCDB collects data annually from tumor registries of Commission on Cancer-accredited programs, comprising approximately 70% of all new US invasive cancer diagnoses. Data collection is standardized based on the Facility Oncology Registry Data Standards (FORDS). For this study, patients diagnosed between 2004–2013 with stages I-III breast, stages I-III colorectal, stages I-III prostate, stages I-II non-small cell lung cancer, stages I-III renal cancers and stages I-II pancreas cancers were identified (S1 Fig). This research was approved (exempted) by the Institutional Review Board of the Taussig Cancer Institute, Cleveland Clinic. We chose the four most common solid tumors amongst men and women; in addition, we included renal and pancreas cancers as less common solid tumors representative of cancers with excellent and poor cure rates, respectively. Patients were excluded if no treatment was given, first treatment occurred > 180 days after diagnosis, interval could not be determined, uncommon histology or uncommon presentations, such as male breast cancer.

### Study definitions

TTI was calculated by NCDB using dates of initial cancer diagnosis and earliest cancer-directed treatment. Date of initial diagnosis was defined as the first date a cancer diagnosis was clinically or histologically established per FORDS definition. Transfer of care was defined as initial treatment received at a facility other than the diagnostic facility. Pathologic American Joint Committee on Cancer (AJCC) stage was used unless unavailable in which case clinical stage was used. Overall survival was measured from date of first treatment to death or last follow-up and was not available for patients diagnosed in 2013. The NCDB censors facility type for all patients under age 40 years (2.2% of patients in our study population). These patients were excluded from the analysis when calculating facility type. We used the Charlson Deyo Index, a modified Charlson comorbidity score based on International Classification of Disease, 9th Edition (ICD-9-CM) coding to assess the severity of preexisting comorbidities (the number of coexisting medical conditions weighted according to their relative effects on survival). The education quartile was derived as follows: educational attainment for each patient's area of residence was estimated by matching the zip code at time of diagnosis against the 2012 American Community Survey data, spanning years 2008¬2012. This provides a measure of the number of adults in the patient's zip code who did not graduate from high school, and is categorized as equally proportioned quartiles amongst all US zip codes.

### Statistical analysis

Statistical analyses evaluated associations between TTI and patient attributes observed at diagnosis, as well as explored TTI for associate with overall survival. Categorical factors were summarized as percentages and continuous factors were summarized as medians and interquartile ranges (IQR). Overall survival is described by the Kaplan-Meier method for various durations of TTI with thresholds of 4, 6, 8 and 10 weeks. Durations of TTI extending beyond 6 weeks are considered delayed in the analysis. The initial statistical analysis plan is provided as a supplemental file (S1 Statistical Analysis Plan).

The Cox proportional hazards model was used for uni- and multiple regression analyses of overall survival. For categorical factors, underlying assumption of proportionality was assessed by plotting log(-log(survival)) x log(time) for each level of the factor and examining the curves for departures from parallelism. For continuous factors the assumption was assessed by plotting Schoenfeld residuals over time, fitting a LOESS line with a 95% confidence band to the points and visually checking to see if the band consistently included a similar approach was used to assess the suitability of using a linear form of these factors in the Cox model; i.e. Martingale residuals were plotted against the factor, a LOESS line with 95% confidence band was fit to the points and the band was visually checked for departures from 0.To account for heterogeneities in clinical protocols by disease staging, separate analyses were performed for each cancer type and stage combination. In addition, to account for any inherent time effects all models were stratified by year of diagnosis.

Univariate hypothesis testing to identify categorical-level factors associated with TTI used Wilxocon rank sum or Kruskal-Wallis tests. Continuous patient attributes were evaluated for correlation with TTI based on the Spearman-rank correlation coefficient. The variance of TTI was generally larger than the mean value suggesting overdispersion and therefore negative binomial regression was used for multiple regression analyses. To obtain the expected mean TTI associated with a particular level of a factor expected mean effects were calculated and averaged across all possible combinations of the other factors in the model holding the specified factor/level fixed. All data analyses were performed using SAS version 9.4 (SAS Institute, Inc, Cary NC).

## Results

### Study population

The study population comprised 3,672,561 patients of whom 37.2% of patients had breast cancer, 25.7% had prostate cancer, 18.0% had colorectal cancer, 9.9% had non-small cell lung cancer, 7.2% had renal cell cancer, and 1.9% had pancreas cancer. Tables 1 and 2 summarize patient characteristics and overall 5-year survival across cancers. The majority of patients (60.0%) were treated at community programs and a third (32.6%) were treated at academic programs. Patients traveled a median (IQR) of 9.4 miles (4.3–21.8) for treatment. Approximately half of patients (51.9%) were insured by government programs.

**Table 1:** Characteristics of the study population by type of cancer.

	All Patients (n = 3,672,561)	Breast (n = 1,368,024)	Prostate (n = 944,246)	Lung (n = 363,863)	Colorectal (n = 662,094)	Renal (n = 262,915)	Pancreas (n = 71,419)
Characteristic	% or Median (IQR)	% or Median (IQR)	% or Median (IQR)	% or Median (IQR)	% or Median (IQR)	% or Median (IQR)	% or Median (IQR)
Sex							
Female	54.9%	100.0%	——	51.1%	49.1%	38.5%	49.4%
Male	45.1%	——	100.0%	49.9%	50.9%	61.5%	50.6%
Race							
White	85.6%	85.5%	85.5%	89.4%	85.7%	85.5%	85.9%
Black	11.3%	11.0%	13.3%	8.4%	10.8%	11.7%	11.0%
Other	3.1%	3.6%	2.5%	2.2%	3.5%	3.2%	3.2%
Age	65 (56–73)	61 (51–71)	65 (59–71)	70 (62–76)	69 (50–79)	62 (53–71)	67 (59–75)
<50	12.7%	21.8%	3.6%	4.3%	9.7%	17.8%	8.4%
50–59	22.2%	24.4%	24.8%	13.8%	18.0%	24.8%	19.0%
60–69	30.2%	25.2%	42.2%	30.8%	23.5%	29.1%	30.3%
70–79	23.4%	18.0%	24.5%	36.1%	26.2%	21.1%	29.5%
≥80	11.5%	10.6%	4.9%	15.0%	22.6%	7.2%	12.9%
Charlson-Deyo Index							
0	77.7%	84.9%	84.3%	54.2%	70.6%	70.3%	67.7%
1	17.3%	12.4%	13.4%	32.5%	21.6%	22.2%	25.2%
>1	5.0%	2.7%	2.3%	13.3%	7.8%	7.5%	7.1%
Insurance							
None	2.2%	2.1%	1.4%	1.7%	3.1%	3.1%	2.7%
Private	46.0%	53.3%	49.8%	27.9%	35.4%	47.7%	37.6%
Government	51.9%	44.6%	48.8%	70.4%	61.4%	49.2%	59.7%
Residence							
Large Urban	52.2%	54.9%	50.1%	49.3%	51.8%	51.1%	52.4%
Small Urban	31.9%	31.1%	32.8%	32.6%	32.1%	31.5%	30.7%
Metropolitan	14.0%	12.4%	15.0%	15.9%	14.1%	15.4%	15.0%
Rural	1.9%	1.6%	2.1%	2.2%	2.0%	2.0%	2.0%

**Table 2:** Overall patient characteristics, time to treatment, and 5-year survival.

	All Patients (n = 3,672,561)	Breast (n = 1,368,024)	Prostate (n = 944,246)	Lung (n = 363,863)	Colorectal (n = 662,094)	Renal (n = 262,915)	Pancreas (n = 71,419)
Characteristic	% or Median (IQR)	% or Median (IQR)	% or Median (IQR)	% or Median (IQR)	% or Median (IQR)	% or Median (IQR)	% or Median (IQR)
Income Quartile							
<38K	16.6%	15.4%	15.8%	19.0%	18.2%	18.0%	17.4%
38-<48K	23.0%	21.9%	22.5%	25.3%	24.2%	24.0%	23.6%
48-<63K	26.9%	26.9%	26.9%	26.9%	27.0%	27.2%	27.0%
≥63K	33.5%	35.8%	35.8%	28.9%	30.7%	30.8%	32.0%
Education (% Not Graduating HS)							
≥21%	15.3%	14.4%	14.2%	16.5%	17.2%	17.5%	15.9%
13–20%	25.0%	23.9%	24.2%	27.7%	26.4%	26.6%	25.8%
7–12%	33.3%	33.3%	33.3%	33.7%	33.2%	32.8%	33.0%
<7%	26.4%	28.4%	28.3%	22.1%	23.7%	23.1%	25.4%
Type of Facility							
Academic/Research	32.6%	20.2%	36.6%	34.7%	26.9%	41.9%	53.4%
Comprehensive Community	60.0%	63.1%	56.4%	58.1%	65.8%	50.7%	39.1%
Integrated Network/Other	7.4%	7.8%	7.0%	7.2%	7.4%	7.4%	7.4%
Distance from Facility (miles)	9.4 (4.3–21.8)	8.5 (4.1–17.9)	11.4 (5.0–27.8)	10.1 (4.4–25.2)	7.9 (3.6–18.0)	12.0 (5.2–31.0)	14.2 (5.7–39.7)
≤10	52.2%	56.3%	45.9%	50.0%	58.2%	44.3%	40.0%
>10–20	20.8%	21.8%	21.0%	19.4%	19.3%	20.3%	19.0%
>20–30	9.0%	8.6%	9.9%	9.7%	7.9%	9.8%	10.0%
>30–40	4.9%	4.3%	5.5%	5.9%	4.3%	5.9%	6.2%
>40–50	3.0%	2.4%	3.4%	3.7%	2.6%	4.0%	4.4%
>50	10.2%	6.6%	14.3%	11.3%	7.7%	15.7%	20.4%
First Cancer							
No	15.8%	16.1%	7.7%	29.2%	18.2%	18.1%	17.1%
Yes	84.2%	84.9%	92.3%	70.8%	81.8%	81.9%	82.9%
Stage							
I	41.1%	54.2%	8.5%	77.0%	30.0%	73.4%	23.8%
II	44.4%	34.1%	80.5%	23.0%	35.1%	11.2%	76.2%
III	14.5%	11.7%	11.0%	——	34.7%	15.4%	——
Transfer of Care							
No	85.6%	81.3%	85.6%	87.1%	89.9%	96.9%	77.7%
Yes	14.4%	18.7%	14.4%	12.9%	10.1%	3.1%	22.3%
Year of Diagnosis							
2004–2005	18.5%	17.5%	19.9%	17.8%	20.3%	16.3%	14.4%
2006–2007	20.2%	18.9%	22.8%	19.5%	20.4%	18.8%	17.2%
2008–2009	20.6%	20.2%	21.6%	20.1%	20.1%	20.7%	19.6%
2010–2011	20.5%	21.0%	20.0%	20.8%	19.5%	21.5%	22.7%
2012–2013	20.3%	22.5%	15.8%	21.7%	19.6%	22.7%	22.1%
Time to Treatment (days)	27 (7–50)	24 (13–38)	57 (31–87)	29 (6–52)	10 (0–27)	0 (0–34)	20 (3–36)
5-Year Survival	77% ± .02%	85% + 0.04%	89% + 0.04%	47% + 0.1%	65% + 0.07%	80% + 0.1%	20% + 0.2%
Follow-up Period (months)	——	56 (0–133)	57 (0–132)	45 (0–133)	53 (0–133)	50 (0–132)	34 (0–130)

### Trends in TTI

**Fig 1:**
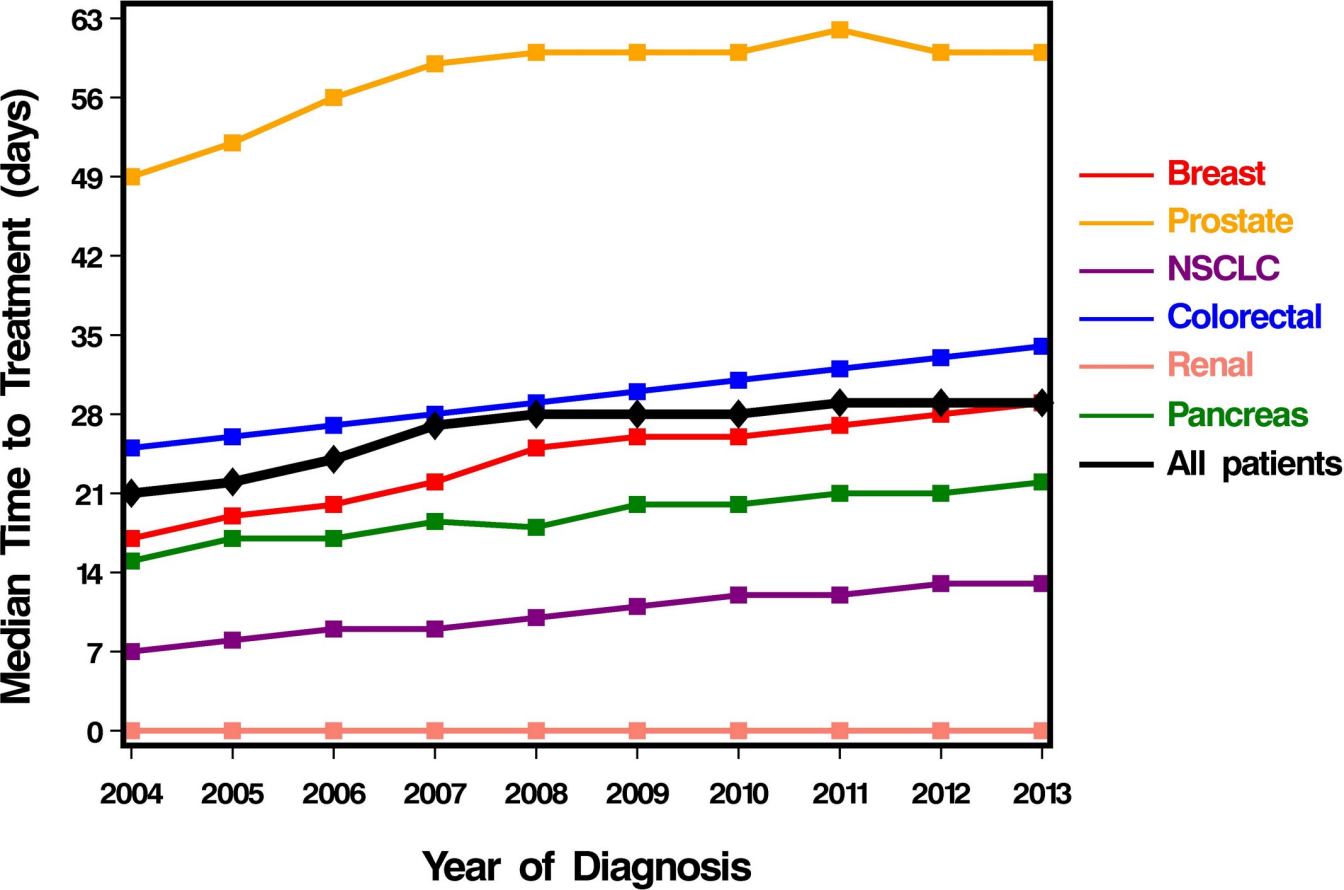
Trends in time to treatment initiation over study period. TTI increased significantly for all cancers from an overall median of 21 days in 2004–2005 to a median of 29 days in 2013–2014 (P<0.001).

The overall median TTI was 27 days. TTI increased significantly across various cancers over study duration (Fig 1, S1 Table), from an overall median of 21 days in 2004 to a median of 29 days in 2013 (P<0.001). Cancers with the greatest increase in median TTI included breast and prostate [absolute increase of 10 days for each—breast: 18 to 28 days (55.6% relative increase); prostate: 50 to 60 days (20.0% relative increase)]. Increase in median TTI was also seen in non-small cell lung (absolute increase of 8 days, relative increase 30.8%), pancreas (absolute increase of 7 days, relative increase 46.7%), and colorectal cancers (absolute increase of 6 days, relative increase 86%). Only median TTI for renal cancer remained stable at 0 days, although even here patients with stages II and III disease saw median increases of 5 and 9 days, respectively.

### Predictors of increased TTI

Multivariable analyses of clinical and socioeconomic factors that could potentially be associated with TTI are summarized in Tables 3 and 4. Several predictors of increased TTI were common to the cancers studied particularly care at academic center, race, education, prior history of cancer, transfer of care, significant comorbidities and age. Treatment at an academic center was associated with increased TTI across all cancers (estimated mean increase of 8.8 days for renal, 5.7 for prostate, 5.5 for colorectal, 4.2 for NSCLC, 4.1 for breast, and 2.0 for pancreas cancers). Black race was associated with increased TTI compared to Whites, ranging from an estimated increase of less than one day for colorectal cancer to 6.7 days for non-small cell lung cancer. Transfer of facility was generally associated with increased TTI, which ranged from an estimated mean of 6.2 days for colorectal, 7.0 for pancreas, and 11.1 for non-small cell lung cancer, to 21.7 for renal cancer.

**Table 3:** Multivariable analyses of predictors of increased time to treatment initiation^a^^,^ ^b^.

	Breast			Prostate			Lung		
Factor	Relative Effect (95% C.I.)	Estimated Effect in Days	p	Relative Effect (95% C.I.)	Estimated Effect in Days	p	Relative Effect (95% C.I.)	Estimated Effect in Days	p
Sex									
Female	**—-**	**—-**	**—-**	**—-**	**—-**	**—-**	Reference	**—-**	**—-**
Male	**—-**	**—-**	**—-**	**—-**	**—-**	**—-**	1.01 (1.00–1.02)	0.6	0.03
Race									
White	Reference	**—-**	**—-**	Reference	**—-**	**—-**	Reference	**—-**	**—-**
Black	1.13 (1.13–1.14)	3.8	<0.001	1.08 (1.07–1.09)	3.5	<0.001	1.16 (1.13–1.18)	6.7	<0.001
Other	1.05 (1.04–1.06)	1.4	<0.001	1.02 (1.01–1.04)	1	<0.001	1.04 (1.01–1.08)	1.7	0.04
Age (per decade)^c^	0.99 (0.99–0.99)	-0.3	<0.001	0.91 (0.90–0.91)	-4.4	<0.001	1.08 (1.07–1.09)	2.9	<0.001
Charlson-Deyo Index									
0	Reference	—-	—-	Reference	—-	—	Reference	—-	—-
1	1.03 (1.02–1.04)	1	<0.001	0.94 (0.94–0.95)	-2.9	<0.001	0.98 (0.97–0.99)	-0.9	0.001
>1	1.08 (1.07–1.09)	2.3	<0.001	0.87 (0.86–0.88)	-6.5	<0.001	1.05 (1.04–1.07)	2.4	<0.001
Insurance									
None	Reference	—-	—-	Reference	—-	—-	Reference	—-	—-
Private	0.85 (0.83–0.86)	-5	<0.001	1.00 (0.98–1.02)	-0.2	0.97	0.81 (0.78–0.85)	-9.3	<0.001
Government	0.90 (0.89–0.91)	-1.7	<0.001	1.00 (0.98–1.02)	-0.9	0.83	0.91 (0.88–0.95)	-4.4	<0.001
Residence									
Large Urban	Reference	—-	—-	Reference	—-	—-	Reference	—-	—-
Small Urban	0.91 (0.91–0.91)	-3	<0.001	0.99 (0.98–0.99)	-0.6	<0.001	1.05 (1.04–1.06)	2.1	<0.001
Metropolitan	0.83 (0.82–0.84)	-5.7	<0.001	0.93 (0.92–0.94)	-3.3	<0.001	1.05 (1.03–1.07)	2.1	<0.001
Rural	0.79 (0.77–0.80)	-7.2	<0.001	0.90 (0.88–0.91)	-5	<0.001	1.05 (1.01–1.09)	2.3	0.02
Income (per quartile)	1.01 (1.01–1.01)	0.2	<0.001	1.02 (1.02–1.03)	1	<0.001	0.97 (0.96–0.98)	-1.5	<0.001
Education (per quartile)	0.97 (0.97–0.98)	-0.9	<0.001	1.00 (0.99–1.00)	-0.2	<0.001	0.98 (0.97–0.99)	-1	<0.001
Type of Facility									
Academic/Research	Reference	—-	—-	Reference	—-	—-	Reference	—-	—-
Comprehensive Community	0.87 (0.87–0.88)	-4.1	<0.001	0.88 (0.88–0.89)	-5.7	<0.001	0.91 (0.90–0.92)	-4.2	<0.001
Integrated Network/Other	0.95 (0.94–0.96)	-1.6	<0.001	0.93 (0.92–0.93)	-3.6	<0.001	0.93 (0.92–0.95)	-3.2	<0.001
Distance from Facility (miles)^d^	1.01 (0.01–1.01)	0.3	<0.001	1.02 (1.02–1.03)	1.1	<0.001	0.98 (0.98–0.99)	-0.8	<0.001
Cancer History									
Yes	Reference	—-	—-	Reference	—-	—-	Reference	—-	—-
No	1.02 (1.01–1.02)	0.4	<0.001	0.77 (0.77–0.78)	-11.8	<0.001	1.01 (1.00–1.02)	0.6	0.04
Stage									
I	Reference	—-	—-	Reference	—-	—-	Reference	—-	—-
II	1.01 (1.01–1.02)	0.4	<0.001	1.10 (1.09–1.11)	4.3	<0.001	1.04 (1.03–1.05)	1.7	<0.001
III	0.98 (0.97–0.98)	-0.7	<0.001	1.05 (1.04–1.06)	2	<0.001	—-	—-	—-
Transfer of Care									
No	Reference	—-	—-	Reference	—-	—-	Reference	—-	—-
Yes	1.00 (0.99–1.00)	0.1	0.21	0.76 (0.76–0.77)	-12.4	<0.001	1.28 (1.26–1.30)	11.1	<0.001

^a^ For each cancer all factors listed were included in the multivariable model

^b^ For categorical factors the data presented are the relative and absolute changes in the expected time to treatment compared to the reference group. For ordinal/continuous the data are the relative and absolute changes associated with a one unit increase.

^c^ <50, 50–59, 60–69, 70–70, > 80

^d^ Distance from the patient’s home to the treating facility in 10 mile increments up to 50, then >50 as a group

**Table 4:** Multivariable analyses of predictors of increased time to treatment initiation^a^^,^ ^b^.

	Colorectal			Renal			Pancreas		
Factor	Relative Effect (95% C.I.)	Estimated Effect in Days	p	Relative Effect (95% C.I.)	Estimated Effect in Days	p	Relative Effect (95% C.I.)	Estimated Effect in Days	p
Sex									
Female	Reference	—-	—-	Reference	—-	—-	Reference	—-	—-
Male	1.09 (1.08–1.10)	1.7	<0.001	1.01 (0.99–1.04)	0.5	0.21	1.01 (0.99–1.03)	0.3	0.35
Race									
White	Reference	—-	—-	Reference	—-	—-	Reference	—-	—-
Black	1.04 (1.03–1.06)	0.8	<0.001	1.07 (1.03–1.11)	2.3	<0.001	1.12 (1.08–1.16)	3.1	<0.001
Other	1.07 (1.04–1.09)	1.3	<0.001	1.00 (0.94–1.07)	0.1	0.95	0.99 (0.93–1.06)	0.2	0.85
Age (per decade)^c^	1.00 (1.00–1.00)	<0.1	0.95	1.05 (1.04–1.07)	1.6	<0.001	1.06 (1.05–1.07)	1.5	<0.001
Charlson-Deyo Index									
0	Reference	—-	—-	Reference	—-	—-	Reference	—-	—-
1	1.02 (1.01–1.04)	0.5	<0.001	1.08 (1.06–1.12)	2.7	<0.001	1.03 (1.00–1.05)	0.7	0.05
>1	1.03 (1.01–1.05)	0.6	<0.001	1.23 (1.18–1.29)	7.3	<0.001	1.07 (1.03–1.12)	2	0.001
Insurance									
None	Reference	—-	—-	Reference	—-	—-	Reference	—-	—-
Private	1.06 (1.03–1.09)	1.2	<0.001	0.78 (0.73–0.83)	-8.6	<0.001	0.96 (0.89–1.04)	-1	0.31
Government	1.10 (1.07–1.12)	1.8	<0.001	0.88 (0.82–0.94)	-4.7	<0.001	1.01 (0.94–1.09)	0.4	0.73
Residence									
Large Urban	Reference	—-	—-	Reference	—-	—-	Reference	—-	—-
Small Urban	0.94 (0.93–0.95)	-1.3	<0.001	1.03 (1.00–1.06)	1.1	0.01	1.01 (0.98–1.04)	0.4	0.37
Metropolitan	0.93 (0.91–0.94)	-1.6	<0.001	1.00 (0.96–1.04)	<0.1	0.98	0.98 (0.94–1.02)	-0.6	0.34
Rural	0.85 (0.82–0.88)	-3.2	<0.001	1.01 (0.93–1.10)	0.4	0.75	0.92 (0.85–1.01)	-2.1	0.09
Income (per quartile)	1.00 (0.99–1.01	<0.1	0.87	0.98 (0.96–0.99)	-1	<0.001	0.99 (0.98–1.01)	-0.2	0.25
Education (per quartile)	0.98 (0.98–0.99)	-0.3	<0.001	0.99 (0.98–1.01)	-0.2	0.41	0.99 (0.97–1.00)	-0.4	0.08
Type of Facility									
Academic/Research	Reference	—-	—-	Reference	—-	—-	Reference	—-	—-
Comprehensive Community	0.76 (0.75–0.77)	-5.5	<0.001	0.78 (0.76–0.80)	-8.8	<0.001	0.93 (0.91–0.96)	-2	<0.001
Integrated Network/Other	0.82 (0.81–0.84)	-4	<0.001	0.86 (0.82–0.90)	-5.7	<0.001	0.84 (0.80–0.87)	-4.8	<0.001
Distance from Facility (miles)^d^	1.03 (1.03–1.04)	0.7	<0.001	1.00 (0.99–1.01)	0.1	0.63	1.00 (0.99–1.01)	<0.1	0.62
Cancer History									
es	Reference	—-	—-	Reference	—-	—-	Reference	—-	—-
No	1.08 (1.07–1.09)	1.5	<0.001	1.23 (1.20–1.27)	7.3	<0.001	1.07 (0.03–1.10)	1.7	<0.001
Stage									
I	Reference	—-	—-	Reference	—-	—-	Reference	—-	—-
II	1.06 (1.05–1.07)	1.2	<0.001	0.80 (0.77–0.83)	-7.8	<0.001	0.86 (0.83–0.88)	-4.2	<0.001
III	1.04 (1.02–1.05)	0.7	<0.001	0.83 (0.80–0.86)	-6.7	<0.001	—-	—-	—-
Transfer of Care									
No	Reference	—-	—-	Reference	—-	—-	Reference	—-	—-
Yes	1.38 (1.36–1.40)	6.2	<0.001	1.92 (1.79–2.05)	21.7	<0.001	1.30 (1.26–1.33)	7	<0.001

^a^ For each cancer all factors listed were included in the multivariable model

^b^ For categorical factors the data presented are the relative and absolute changes in the expected time to treatment compared to the reference group. For ordinal/continuous the data are the relative and absolute changes associated with a one unit increase.

^c^ <50, 50–59, 60–69, 70–70, > 80

^d^ Distance from the patient’s home to the treating facility in 10 mile increments up to 50, then >50 as a group

### Association of TTI with survival

**Fig 2:**
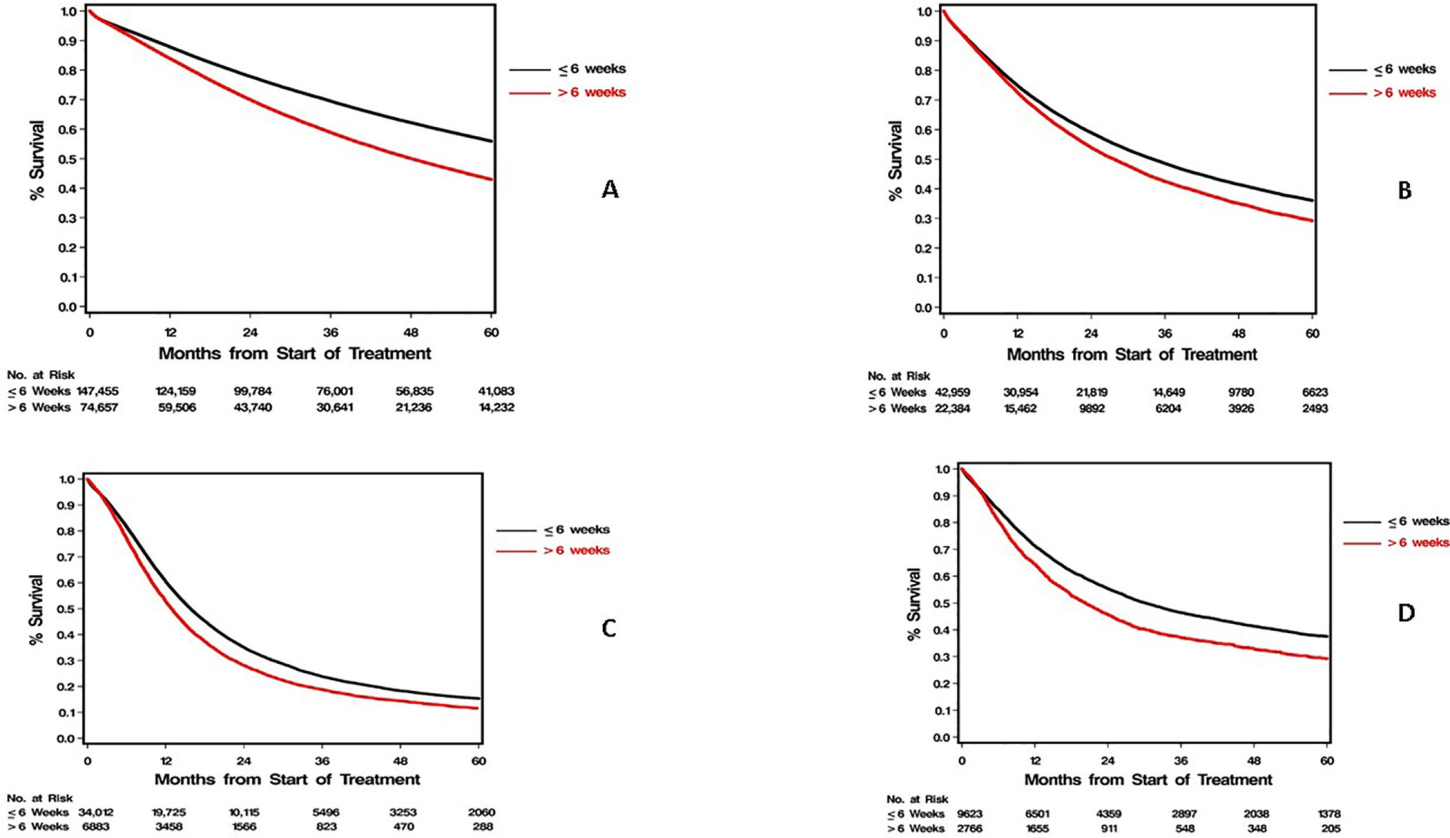
Overall survival by prolonged treatment delay in stages I and II non-small cell lung and pancreas cancers. Five-year overall survival for National Cancer Database patients with time to treatment initiation of six weeks or less was substantially higher when compared to patients with time to treatment initiation greater than six weeks for stage I (A) and stage II (B) non-small cell lung cancer and stage I (C) and stage II (D) pancreas cancers (P<0.001 for each).

Multivariable analyses of the association of TTI with overall survival from start of treatment (measured in weeks) after stratification by diagnosis year (in two-year intervals) and adjustment for baseline clinical and sociodemographic factors, are summarized in Table 5. For the majority of cancer sites and stages studied, increased TTI was associated with worsened survival with the exceptions of stages I-III prostate and stages II-III colorectal cancers. The largest association was seen in pancreas and non-small cell lung cancer. Every week of increased TTI was associated with increased risk of death by an estimated 3.0% and 2.4% in stage I and II pancreas cancer, and 3.2% and 1.6% in stage I and II non-small cell lung cancer, respectively. Increased TTI also associated with worsened survival in stages I and II breast and renal cancers. In stage I breast and renal cell cancer the risks increased an estimated 1.8% and 1.2% per week, respectively; and in stage II disease by 1.2% in both. Increased TTI was associated to a lesser degree with outcomes in stage III breast and renal cell cancer (0.1% and 0.3% estimated increase in risk per week, respectively), and stage I colorectal cancer (0.5% increase per week). Prolonged TTI (> 6 weeks) was associated with substantial worsening of survival across all cancers with the exception of prostate (S2 Table). The most substantial associations with worsened mortality were seen in patients with lung and pancreas cancers. Five-year overall survival for stage I NSCLC was 56% (±0.2) for TTI ≤ 6wks compared to 43% (±0.2) for TTI > 6 wks and for stage I pancreas was 38% (±0.6) v 29% (±1) respectively (P<0.001 for both) (Fig 2, S2 Fig).

**Table 5:** Multivariable analyses of the association of increased TTI with overall survival^a^.

	Stage I		Stage II		Stage III	
Cancer	Hazard Ratio (95% C.I.)^b^	p	Hazard Ratio (95% C.I.)^b^	p	Hazard Ratio (95% C.I.)^b^	p
Breast	1.018 (1.015–1.020)	<0.001	1.012 (1.010–1.015)	<0.001	1.001 (0.998–1.004)	0.72
Prostate	0.941 (0.936–0.947)	<0.001	0.969 (0.968–0.970)	<0.001	0.964 (0.960–0.969)	<0.001
NSCLC	1.032 (1.031–1.034)	<0.001	1.016 (1.014–1.018)	<0.001	—-	—-
Colorectal	1.005 (1.002–1.008)	<0.001	0.981 (0.978–0.984)	<0.001	0.971 (0.968–0.974)	<0.001
Renal	1.013 (1.010–1.015)	<0.001	1.012 (1.006–1.019)	<0.001	1.003 (0.999–1.008)	0.16
Pancreas	1.030 (1.025–1.035)	<0.001	1.024 (1.021–1.027)	<0.001	—-	—-

^a^ All models were stratified by year of diagnosis; other factors in the model included gender (except breast and prostate), age, race, Charlson-Deyo index, insurance status, type of facility, distance from reporting facility, income, education, residence, whether or not this is the patient’s first cancer, and if treatment was administered at the reporting facility. Tumor site (colon, rectum, rectosigmoid junction) was included in the analysis of colorectal cancer

^b^ Hazard ratios >1 indicate the risk of death increases as the treatment delay increases; ratios <1 that the risk decreases.

In order to address unmeasured confounding arising from association between patient prognosis and treatment pathway, the associations identified between TTI and clinical outcomes were re-evaluated in subgroup analyses adjusting for treatment selection. When analysis was restricted to patients receiving surgery as first-line therapy in patients with breast and lung cancer, the standard of care for these populations, statistical estimators demonstrated stronger effect sizes and maintained statistical significance with p-values not exceeding 0.001(Table 6).

**Table 6:** Analysis of the association of increased TTI with overall survival in patients receiving surgery alone as first treatment for breast and lung cancers.

	Stage I		Stage II	
Cancer Type	Hazard Ratio	P-value	Hazard Ratio	P-value
Breast	1.017 (1.014–1.020)	<0.001	1.006 (1.003–1.009)	0.0002
Lung	1.024 (1.022–1.026)	<0.001	1.017 (1.014–1.021)	<0.001

## Discussion

We conducted a comprehensive analysis of TTI across a variety of common solid tumors and found a substantial worsening of TTI over recent years. Increased TTI was associated with increased risk of mortality ranging from 1.2–3.2% per week in early-stage breast, lung, renal and pancreas cancers.

A major finding of our analysis is worsening TTI over the years of study 2004–13, with a relative worsening of 38% across all cancers studied (range, 20% to 86%). Our findings are consistent with a prior analysis that also demonstrated an increased TTI over the years 1995–2005 but only evaluated surgery as first treatment [20]. Our analysis extends this by showing persistent worsening TTI, even when all types of initial treatment are included. Prior authorizations of diagnostic and therapeutic modalities are increasingly being mandated [18, 19], but we did not have access to direct or surrogate markers of this process to determine if such processes impacted on TTI. The benefits, if any, of such mandates have not been fully studied and our findings suggest the potential of harm to patients [16]. Notably, in our analysis, uninsured patients who do not have to go through a prior authorization process had a faster TTI.

There were common predictors of increased TTI across various cancers studied. A particularly surprising finding of our analysis was the association of lengthened TTI with treatment at an academic program. This may be a reflection of the complexity, acuity, racial profile and insurance status of patients seen at academic centers, although our findings were adjusted for these variables. Much is known about health care disparities by race in the US [22, 23, 24, 25], and our finding that Black race is associated with increased TTI is discouraging but not unexpected. Interestingly, uninsured patients had a faster TTI. It is possible that because uninsured patients do not have to go through prior authorization processes, TTI is somewhat abbreviated. We note the heterogeneity in TTI in prostate cancer in particular. We suspect these findings may be explained by the use of active surveillance in low-risk patients, whose treatment may be delayed for months or years and who are usually cured at that timepoint, as opposed to those who are treated quickly because they have more aggressive disease and a correspondingly higher chance of relapse. The NCDB does not have all the physician and patient factors that go into decision-making about treatment versus surveillance in prostate cancer so we cannot explore this hypothesis statistically.

Perhaps the most concerning finding in our analysis is the association of increased TTI with increased risk of mortality. Prolonged TTI of over 6 weeks was associated with a 13% absolute increase in 5-year mortality in stage I non-small cell lung cancer and a 9% absolute increase in stage I pancreas cancer. Our findings are consistent with prior reports demonstrating an association of worsened mortality with increased time to surgery in early stage breast cancer [5] and in patients with lung cancer with TTI over 35 days [8]. Our findings were consistent across the majority of cancers studied, with the exception of prostate cancer which has a well-known indolent natural history. Of note, the association with survival is more emphatic in earlier stages of the cancers studied; this may be reflective of lower use of systemic therapy in those settings [26]. In the context of findings of potential harm, we believe it is incumbent upon insurers and health systems to simplify access and reduce operational barriers to initiating treatment. Recent initiatives have shown that TTI can be substantially reduced by multidisciplinary approaches and cost-neutral interventions and such efforts need to be adopted more broadly and with a greater sense of urgency [27, 28].

There are certainly limitations to our analysis. This was a retrospective cohort study, although the NCDB includes a majority of cancer patients treated in the US and therefore provides assurance of external validity. We could not account for non-standard-of-care treatments or experimental agents, although the latter constitute < 1% of treatments provided in this dataset. To control for confounding arising from heterogeneity with respect to non-standard-of-care treatment pathways, we implemented a subgroup analysis restricted to patients receiving surgery in first line therapy for breast and lung cancer, the standard of care for these populations. Characterizing the impact of TTI for an average patient, the subgroup analysis demonstrated stronger association between survival and TTI which would reduce the likelihood that treatment variation or non-standard treatment plays an important role in the association of TTI with survival. Although we adjusted for available variables associated with mortality and conducted separate multivariate analyses for each site and stage of cancer, we cannot account for unmeasured confounders of TTI and cancer outcomes. A randomized comparison of early versus delayed treatment would represent an ideal study design but would not be acceptable to patients and be likely considered unethical. Finally, we could not account for insurance authorization processes or patient preferences leading to delays in initiating treatment.

The findings of potential harm associated with increased TTI suggest that studies of interventions designed to simplify access and navigation are warranted. Recent trials have shown that patient navigation can have a moderate benefit in assuring timely cancer care [29, 30, 31]. Prior authorization processes for medications may contribute to delay in medication receipt [32, 33]. The multidisciplinary nature of modern cancer care may introduce new inefficiencies; team-based approaches have been shown to reduce time to treatment in colorectal cancer, for instance, by as much as a third [26, 27]. Such processes need to be adopted more widely.

In our analysis of six early-stage solid tumors treated in curative settings, TTI has lengthened substantially over recent years. Increased TTI is associated with increased risk of death in early-stage breast, lung, renal and pancreas cancers. We believe that studies of interventions to simplify access, ease navigation and reduce barriers are warranted to diminish the potential harm to patients.

## Supporting information

S1 STROBE Checklist Cohort study. A checklist of items to be included in articles reporting observational research.
(DOCX)

S1 Statistical Analysis Plan A technical and detailed elaboration of the principal features of the statistical analysis described in the methods. (DOCX)

S1 Table Changes in time to treatment over time (days) by type of cancer and stage. Table describing absolute and relative change in the median days to treat over time by disease group.
(DOCX)

S2 Table Overall survival at 5-years in patients with treatment delays ≤6 weeks versus >6 weeks. Kaplan-Meier estimates of 5-year overall survival for each cancer type reported by stage. Estimates are stratified by TTI duration threshold of sic weeks. 95% confidence interval were computer using Greenwood’s formula.
(DOCX)

S3 Table Characteristics of first treatment type for lung and breast cancers. Table describing the population of patients where surgical treatment was chosen in the first line setting for lung and breast cancer patients with stage I and II disease.
(DOCX)

S1 Fig Consort diagram. Flowchart of patients from the National Cancer Database included for analysis of time to treatment initiation and overall survival, after evaluation for eligibility criteria.
(TIF)

S2 Fig Overall survival by prolonged treatment delay in stages I and II non-small cell lung and pancreas cancers- 4, 8, and 10 week models. Time to treatment initiation was modeled as a continuous variable in Cox models. To illustrate the effect of time of treatment initiation at various timepoints, plots at 4 weeks (2A and 2D), 8 weeks (2B and 2E), and 10 weeks (2C and 2F) demonstrate that five-year overall survival for National Cancer Database patients for stage I and II non-small cell lung cancer and stage I and II pancreas cancers (P <0.001 for each).
(TIF)

S3 Fig Pairwise Spearman correlation matrix between time to treatment initiation factors. Heat map displaying Spearman rank correlations between all pairwise comparisons for factors predicting time to treatment initiation.
(TIF)
