# Correction: Time to initial cancer treatment in the United States and association with survival over time: An observational study

**DOI:** 10.1371/journal.pone.0215108

**Published:** 2019-04-04

**Authors:** Alok A. Khorana, Katherine Tullio, Paul Elson, Nathan A. Pennell, Stephen R. Grobmyer, Matthew F. Kalady, Daniel Raymond, Jame Abraham, Eric A. Klein, R. Matthew Walsh, Emily E. Monteleone, Wei Wei, Brian Hobbs, Brian J. Bolwell

There are errors in the caption for Fig 2, “Overall survival by prolonged treatment delay in stages I and II non-small cell lung and pancreas cancers.” Please see the correct Fig 2 caption below.

**Fig 2:**
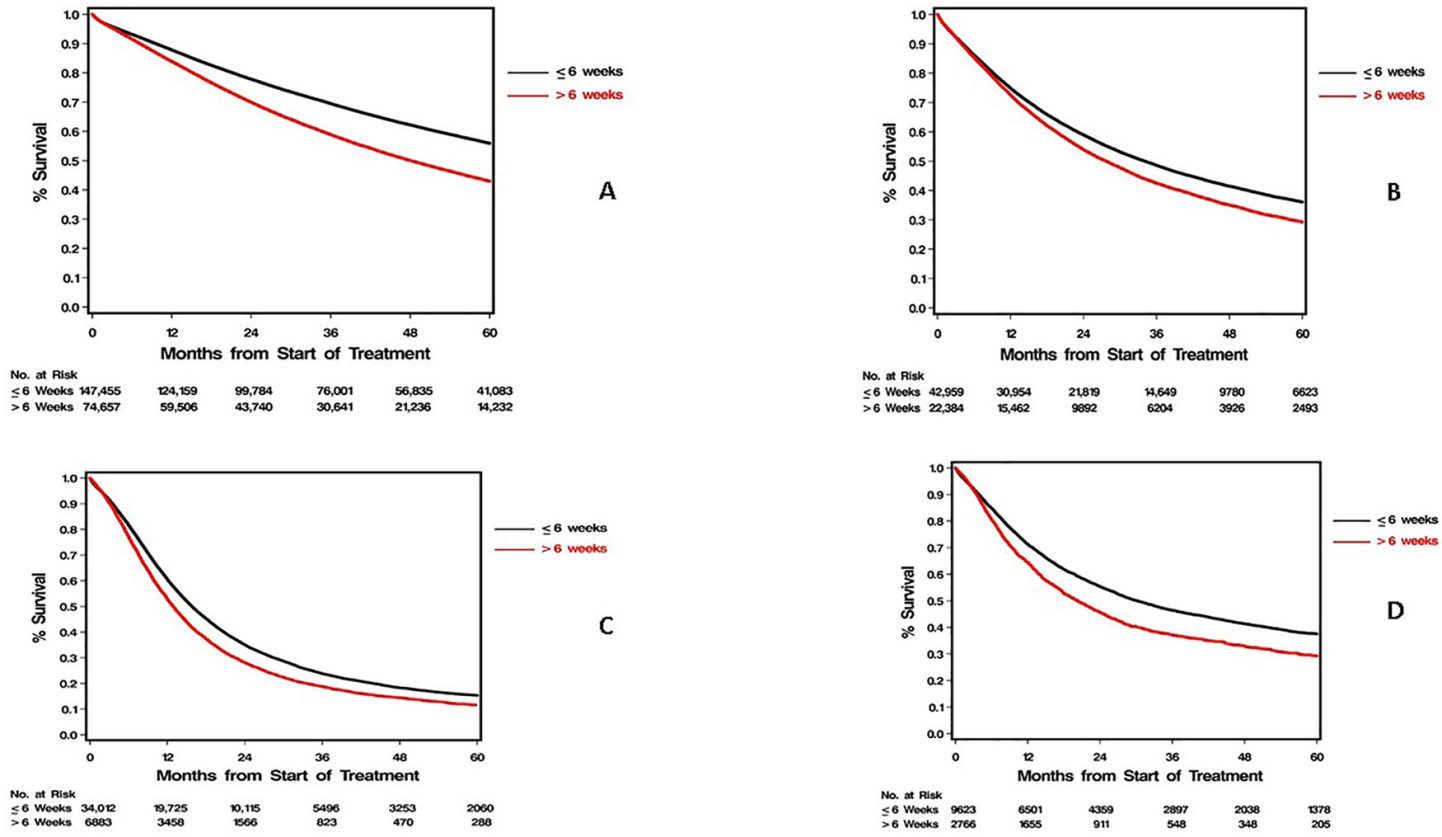
Overall survival by prolonged treatment delay in stages I and II non-small cell lung and pancreas cancers. Five-year overall survival for National Cancer Database patients with time to treatment initiation of six weeks or less was substantially higher when compared to patients with time to treatment initiation greater than six weeks for stage I (A) and stage II (B) non-small cell lung cancer and stage I (D) and stage II (C) pancreas cancers (P<0.001 for each).

There is an error in the last sentence of the second paragraph of the Discussion section. The correct sentence is: Notably, in our analysis, uninsured patients who do not have to go through a prior authorization process had a faster TTI for some cancers.
