# Supplementary material for: Time to initial cancer treatment in the United States and association with survival over time: An observational study
Source: PLoS One. 2019 Mar 1;14(3):e0213209. doi: 10.1371/journal.pone.0213209 (PMC6396925; doi:10.1371/journal.pone.0213209)
Supplement: S1 Statistical Analysis Plan — (DOCX) [file pone.0213209.s002.docx]

**STATISTICAL ANALYSIS PLAN**

Wait times for cancer treatment have been on the rise since 1995, and as cancer incidence continues to rise, wait times are likely to continue to increase. Patients frequently voice concerns regarding wait times to cancer treatment (Bilimoria et. al., 2011). Indeed, 20% to 47% of patients with newly diagnosed and recurrent cancer show a significant level of distress-a risk factor for poorer quality of life, nonadherence to treatment, and increased visits to the physician’s office and emergency room. (NCCN, 2013). Some literature exists about wait times to treatment for cancer patients; however, there are few national data (Bleicher et. al., 2012). Literature that does exist tends to focus on one or a few cancer sites. Further, previous studies largely examine wait times to surgery, and there is a dearth of information regarding times to other treatment modalities. Additionally, there is little information available concerning the relationship between overall survival (OS) and the time elapsed between a patient’s initial diagnosis and first cancer-directed treatment. Delays in time to treatment after diagnosis have been documented to be associated with worse outcomes for some cancers (Landercasper et. al., 2010), but more analysis on the topic is necessary for many cancer sites.

We would like to examine all patients diagnosed between 01/01/2005-12/31/2013. The Fields we are requesting are as follows: Facility Type, Facility Location, Patient Age, Sex, Race, Spanish Origin, Primary Payor, Income 2000, Income 2008-2012, Education 2000, Education 2008-2012, Great Circle Distance, Charlson Score, Sequence Number, Class of Case, Year of Diagnosis, Primary Site, Laterality, Histology, Behavior, Grade, Diagnostic and Staging Procedure (Days from Dx), AJCC Clinical and Pathologic T, N, M, and Stage Groups, TNM Edition Number, Treatment Started-Days from Dx, First Surgical Procedure-Days from Dx, Definitive surgical procedure-Days from Dx, Surgical Procedure of the Primary Site, Radiation-Days from Dx, Radiation Therapy, Location of Radiation Therapy, Radiation Ended-Days from Start of Radiation, Systemic-Days from Dx, Chemotherapy, Chemotherapy-Days from Dx, Hormone Therapy, Hormone Therapy- Days from Dx, Immunotherapy, Immunotherapy-Days from Dx, Systemic Surgery Sequence, Other treatment, Other Treatment-Days from Dx, Last Contact or Death-Months from Dx, PUF Vital Status.

Categorical data such as gender and stage will be summarized as frequency counts and proportions; overall survival will be summarized using the Kaplan-Meier method; other measured factors such as age and time to treatment will be summarized as means and standard deviations or medians and ranges. Time to treatment initiation (TTI) will be summarized using median and range. Negative binomial regression will be used to associate patient characteristics with TTI. Kaplan-Meier method will be used to estimate overall survival by patient group, and log-rank test will be used to compare OS between groups. Cox proportional hazard model will be used to estimate effects of multiple patient characteristics on OS by tumor type, stratified by year of diagnosis. All tests will be two-sided and p-values of 0.05 or less will be considered statistically significant. Statistical analysis will be carried out using SAS version 9.4 (SAS Institute, Cary, NC).
