# Supplementary material for: Time to initial cancer treatment in the United States and association with survival over time: An observational study
Source: PLoS One. 2019 Mar 1;14(3):e0213209. doi: 10.1371/journal.pone.0213209 (PMC6396925; doi:10.1371/journal.pone.0213209)
Supplement: S1 Table — Table describing absolute and relative change in the median days to treat over time by disease group. (DOCX) [file pone.0213209.s003.docx]

**S1 Table. Changes in Time to Treatment Over Time (Days) by Type of Cancer and Stage**

|  |  | **2004-2005** | **2006-2007** | | | **2008-2009** | | | **2010-2011** | | | **2012-2013** | | |
| --- | --- | --- | --- | --- | --- | --- | --- | --- | --- | --- | --- | --- | --- | --- |
| **Cancer Site** | **Stage** | **Median (days)** | **Median** | **Absolute Change^a^** | **Relative Change^a^** | **Median** | **Absolute Change^a^** | **Relative Change^a^** | **Median** | **Absolute Change^a^** | **Relative Change^a^** | **Median** | **Absolute Change^a^** | **Relative Change^a^** |
|  |  |  |  |  |  |  |  |  |  |  |  |  |  |  |
| Breast | I | 18 | 21 | 3 | 17% | 25 | 7 | 39% | 27 | 9 | 50% | 29 | 11 | 61% |
|  | II | 18 | 22 | 4 | 22% | 26 | 8 | 44% | 27 | 9 | 50% | 29 | 11 | 61% |
|  | III | 18 | 21 | 3 | 17% | 24 | 6 | 33% | 25 | 7 | 39% | 26 | 8 | 44% |
| ***Overall*** | | ***18*** | ***21*** | ***3*** | ***17%*** | ***25*** | ***7*** | ***39%*** | ***27*** | ***9*** | ***50%*** | ***28*** | ***10*** | ***56%*** |
| Prostate | I | 4 | 11 | 7 | 175% | 34 | 30 | 750% | 62 | 58 | 1450% | 58 | 54 | 1350% |
|  | II | 51 | 58 | 7 | 14% | 61 | 10 | 20% | 61 | 10 | 20% | 61 | 10 | 20% |
|  | III | 50 | 56 | 6 | 12% | 60 | 10 | 20% | 61 | 11 | 22% | 61 | 11 | 22% |
| ***Overall*** | | ***50*** | ***57*** | ***7*** | ***14%*** | ***60*** | ***10*** | ***20%*** | ***61*** | ***11*** | ***22%*** | ***60*** | ***10*** | ***20%*** |
| Lung | I | 25 | 27 | 2 | 8% | 29 | 4 | 16% | 31 | 6 | 24% | 33 | 8 | 32% |
|  | II | 28 | 30 | 2 | 7% | 32 | 4 | 14% | 33 | 5 | 18% | 35 | 7 | 25% |
| ***Overall*** | | ***26*** | ***28*** | ***2*** | ***8%*** | ***30*** | ***4*** | ***15%*** | ***32*** | ***6*** | ***23%*** | ***34*** | ***8*** | ***31%*** |
| Colorectal | I | 5 | 6 | 1 | 20% | 7 | 2 | 40% | 7 | 2 | 40% | 7 | 2 | 40% |
|  | II | 8 | 10 | 2 | 25% | 12 | 4 | 50% | 13 | 5 | 62% | 14 | 6 | 75% |
|  | III | 8 | 9 | 1 | 12% | 12 | 4 | 50% | 13 | 5 | 62% | 14 | 6 | 75% |
| ***Overall*** | | ***7*** | ***9*** | ***2*** | ***29%*** | ***10*** | ***3*** | ***43%*** | ***12*** | ***5*** | ***71%*** | ***13*** | ***6*** | ***86%*** |
| Renal | I | 0 | 0 | 0 | -0- | 0 | 0 | -0- | 0 | 0 | -0- | 0 | 0 | -0- |
|  | II | 0 | 0 | 0 | -0- | 2 | 2 | --- | 4 | 4 | --- | 5 | 5 | --- |
|  | III | 0 | 0 | 0 | -0- | 3 | 3 | --- | 6 | 6 | --- | 9 | 9 | --- |
| ***Overall*** | | ***0*** | ***0*** | ***0*** | ***-0-*** | ***0*** | ***0*** | ***-0-*** | ***0*** | ***0*** | ***-0-*** | ***0*** | ***0*** | ***-0-*** |
| Pancreas | I | 14 | 17 | 3 | 21% | 20 | 6 | 43% | 24 | 10 | 71% | 25 | 11 | 79% |
|  | II | 15 | 17 | 2 | 13% | 19 | 4 | 27% | 20 | 5 | 33% | 21 | 6 | 40% |
| ***Overall*** | | ***15*** | ***17*** | ***2*** | ***13%*** | ***19*** | ***4*** | ***27%*** | ***20*** | ***5*** | ***33%*** | ***22*** | ***7*** | ***47%*** |
|  | |  |  |  |  |  |  |  |  |  |  |  |  |  |
| ***All Patients*** | | ***21*** | ***25*** | ***4*** | ***19%*** | ***28*** | ***7*** | ***25%*** | ***29*** | ***8*** | ***38%*** | ***29*** | ***8*** | ***38%*** |

^a^ Compared to median during 2004-2005
