# Supplementary material for: Time to initial cancer treatment in the United States and association with survival over time: An observational study
Source: PLoS One. 2019 Mar 1;14(3):e0213209. doi: 10.1371/journal.pone.0213209 (PMC6396925; doi:10.1371/journal.pone.0213209)
Supplement: S2 Table — Kaplan-Meier estimates of 5-year overall survival for each cancer type reported by stage. Estimates are stratified by TTI duration threshold of sic weeks. 95% confidence interval were computer using Greenwood’s formula. (DOCX) [file pone.0213209.s004.docx]

**S2 Table. Overall Survival at 5-Years in Patients with Treatment Delays <6 Weeks versus >6 Weeks^1^**

| **Cancer** | **Delay** | **Stage I** | **Stage II** | **Stage III** |
| --- | --- | --- | --- | --- |
|  |  |  |  |  |
| Breast | ≤6 Weeks | 91% + 0.05% | 84% + 0.1% | 68% + 0.2% |
|  | >6 Weeks | 89% + 0.1% | 82% + 0.2% | 68% + 0.4% |
|  |  |  |  |  |
| Prostate | ≤6 Weeks | 77% + 0.5% | 84% + 0.1% | 86% + 0.2% |
|  | >6 Weeks | 91% + 0.4% | 93% + 0.05% | 93% + 0.1% |
|  |  |  |  |  |
| NSCLC | ≤6 Weeks | 56% + 0.2% | 37% + 0.2% | ---- |
|  | >6 Weeks | 43% + 0.2% | 29% + 0.4% | ---- |
|  |  |  |  |  |
| Colorectal | ≤6 Weeks | 76% + 0.1% | 65% + 0.1% | 57% + 0.1% |
|  | >6 Weeks | 70% + 0.4% | 60% + 0.4% | 57% + 0.4% |
|  |  |  |  |  |
| Renal | ≤6 Weeks | 85% + 0.1% | 79% + 0.3% | 63% + 0.3% |
|  | >6 Weeks | 81% + 0.3% | 71% + 0.9% | 60% + 0.8% |
|  |  |  |  |  |
| Pancreas | ≤6 Weeks | 38% + 0.6% | 15% + 0.2% | ---- |
|  | >6 Weeks | 29% + 1.1% | 12% + 0.5% | ---- |

^1^Kaplan-Meier estimates of 5-year overall survival for each cancer type reported by stage. Estimates are stratified by TTI duration threshold of six weeks. 95% confidence interval were computed using Greenwood’s formula.
