# Supplementary material for: Time to initial cancer treatment in the United States and association with survival over time: An observational study
Source: PLoS One. 2019 Mar 1;14(3):e0213209. doi: 10.1371/journal.pone.0213209 (PMC6396925; doi:10.1371/journal.pone.0213209)
Supplement: S3 Table — Table describing the population of patients where surgical treatment was chosen in the first line setting for lung and breast cancer patients with stage I and II disease. (DOCX) [file pone.0213209.s005.docx]

**S3 Table. Characteristics of First Treatment Type for Lung and Breast Cancers**

| Cancer Type | First Treatment | Stage I | | Stage II | | All | |
| --- | --- | --- | --- | --- | --- | --- | --- |
|  |  | **N** | **%** | **N** | **%** | **N** | **%** |
| Lung | Non-surgery | 58701 | 23.32 | 25979 | 34.47 | 84680 | 25.89 |
|  | Surgery | 193058 | 76.68 | 49386 | 65.53 | 242444 | 74.11 |
| Breast | Non-surgery | 19530 | 2.78 | 55376 | 12.51 | 74906 | 6.54 |
|  | Surgery | 683604 | 97.22 | 387198 | 87.49 | 1070802 | 93.46 |
