# Supplementary figures and images for: Time to initial cancer treatment in the United States and association with survival over time: An observational study
Source: PLoS One. 2019 Mar 1;14(3):e0213209. doi: 10.1371/journal.pone.0213209 (PMC6396925; doi:10.1371/journal.pone.0213209)

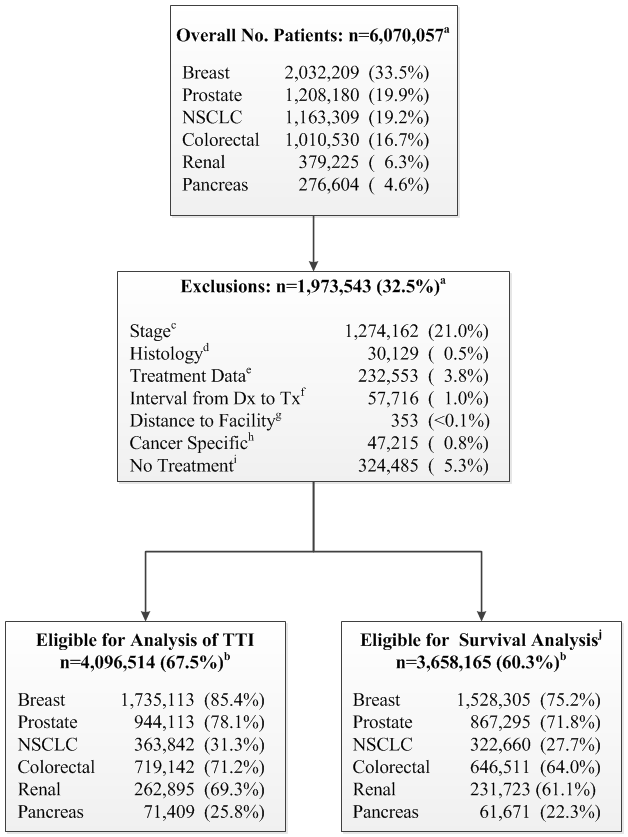

Supplement: S1 Fig — Flowchart of patients from the National Cancer Database included for analysis of time to treatment initiation and overall survival, after evaluation for eligibility criteria. (TIF) [file pone.0213209.s006.tif]

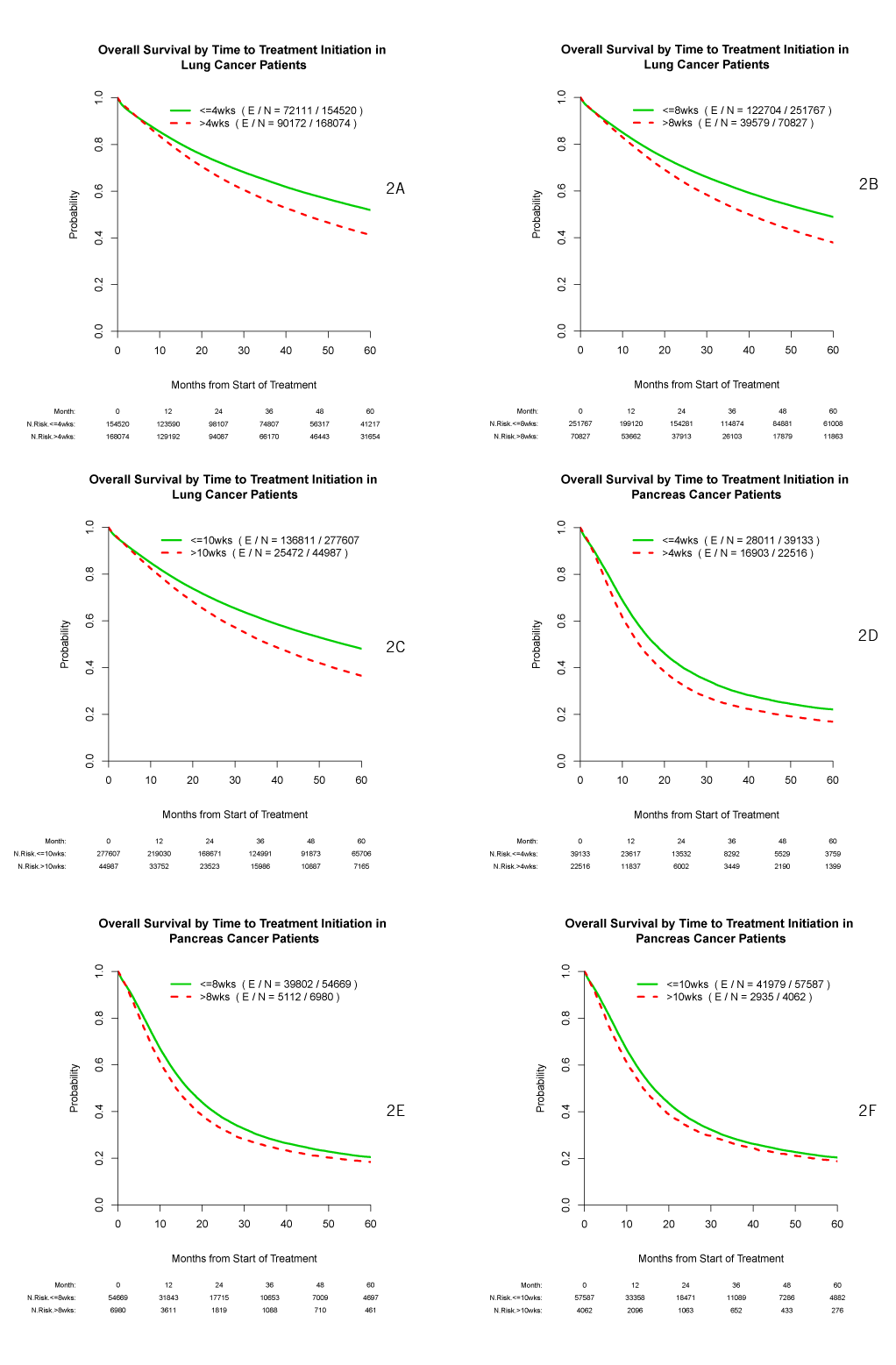

Supplement: S2 Fig — Time to treatment initiation was modeled as a continuous variable in Cox models. To illustrate the effect of time of treatment initiation at various timepoints, plots at 4 weeks (2A and 2D), 8 weeks (2B and 2E), and 10 weeks (2C and 2F) demonstrate that five-year overall survival for National Cancer Database patients for stage I and II non-small cell lung cancer and stage I and II pancreas cancers (P <0.001 for each). (TIF) [file pone.0213209.s007.tif]

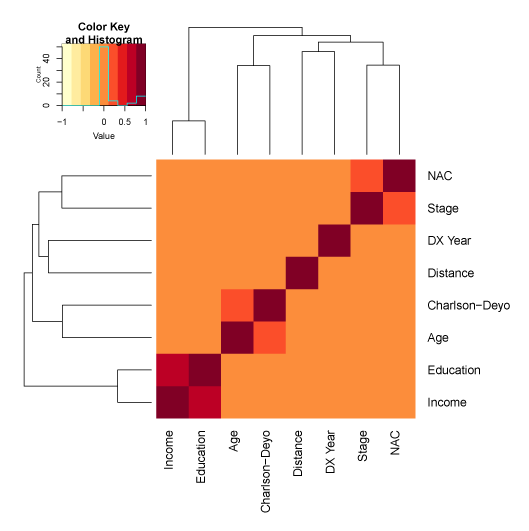

Supplement: S3 Fig — Heat map displaying Spearman rank correlations between all pairwise comparisons for factors predicting time to treatment initiation. (TIF) [file pone.0213209.s008.tif]
